# Supplementary material for: Optical emissions associated with narrow bipolar events from thunderstorm clouds penetrating into the stratosphere
Source: Nat Commun. 2021 Nov 17;12:6631. doi: 10.1038/s41467-021-26914-4 (PMC8599706; doi:10.1038/s41467-021-26914-4)
Supplement: Supplementary file 1 — Supplementary Information [file 41467_2021_26914_MOESM1_ESM.pdf]

Supplementary information

Optical emissions associated with narrow bipolar events from thunderstorm clouds penetrating into the stratosphere

Feifan Liu[1], Gaopeng Lu[1], Torsten Neubert[2], Jiuhou Lei[1,3*], Oliver Chanrion[2], Nikolai Østgaard[4], Dongshuai Li[5], Alejandro. Luque[5], Francisco J. Gordillo-Vázquez[5], Victor Reglero[6], Weitao Lyu[7] , and Baoyou Zhu[1*]

1. CAS Key Laboratory of Geospace Environment, School of Earth and Space Sciences, University of Science and Technology of China, Hefei, China.

2. National Space Institute, Technical University of Denmark (DTU Space), Kongens Lyngby, Denmark.

3. CAS Center for Excellence in Comparative Planetology, University of Science and Technology of China, Hefei, China

4. Birkeland Centre for Space Science, Department of Physics and Technology, University of Bergen, Norway.

5.Instituto de Astrofísica de Andalucía, CSIC, Glorieta de la Astronomia s/n, Granada, Spain.

6. Image Processing Laboratory, University of Valencia, Valencia, Spain.

7. State Key Laboratory of Severe Weather, Chinese Academy of Meteorological Sciences, Beijing, China.

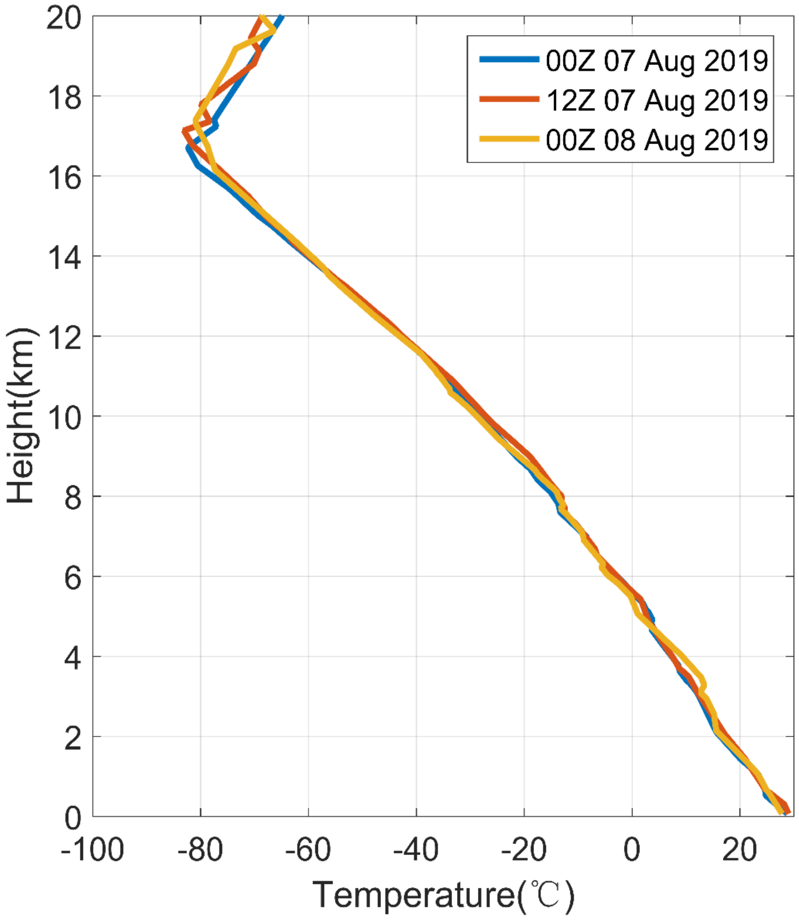

Supplementary Figure 1 | The radiosonde temperature obtained from HongKong sounding station (114.17°E, 22.31°N) close to the NBE producing thunderstorm.

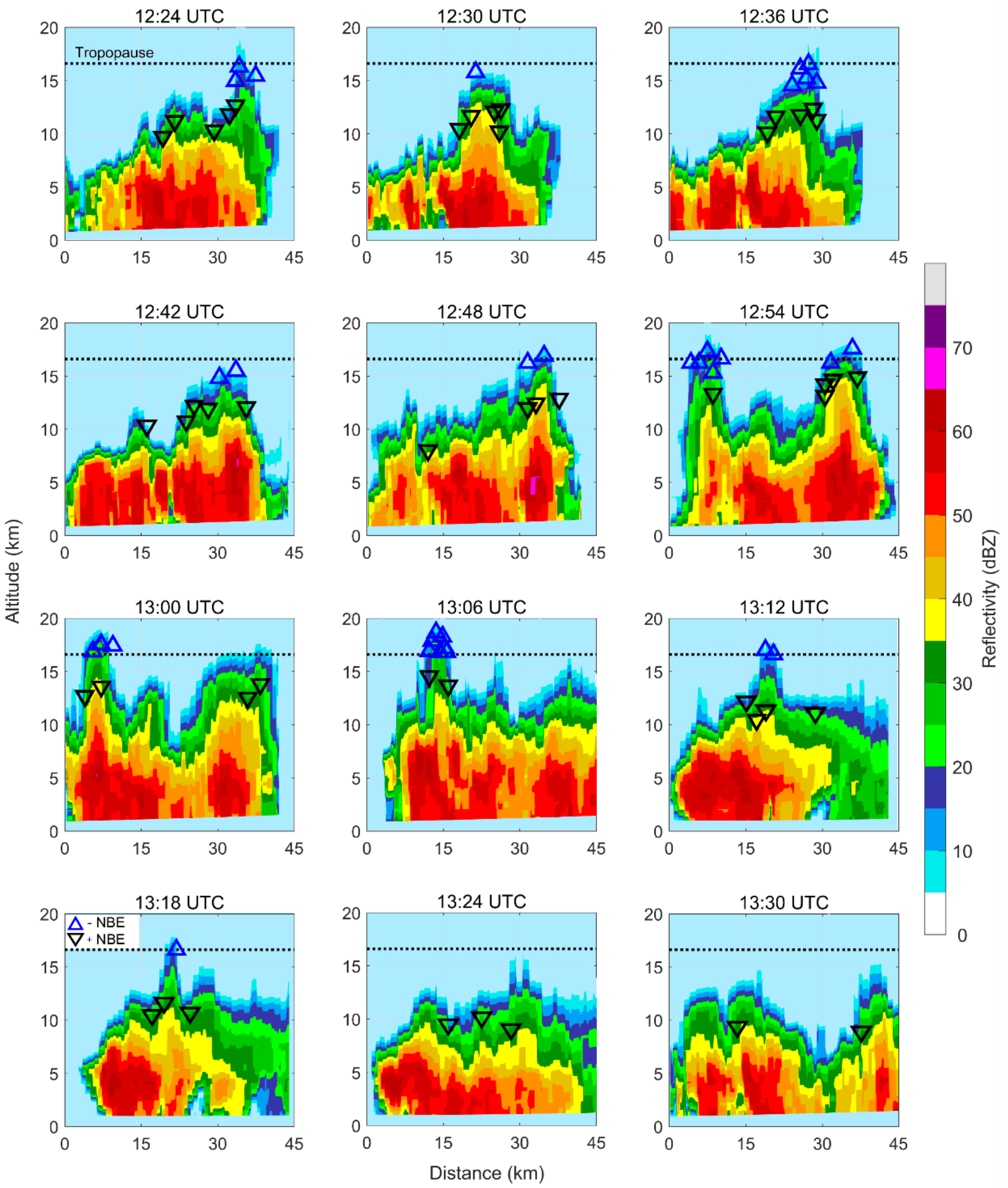

Supplementary Figure 2 | Evolution of the radar reflectivity through the parent thunderstorm. The blue '△' represents the negative NBEs. The black '▽' presents the positive NBEs.

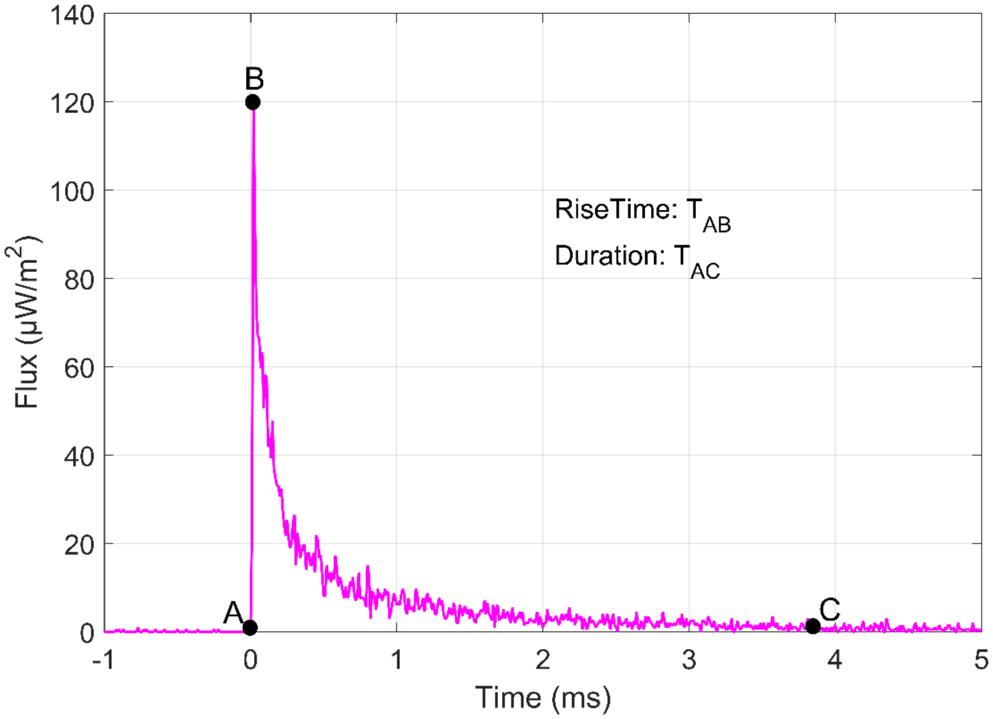

Supplementary Figure 3 | Definition of the waveform parameters for the blue emission events. Point A present the beginning time of the blue emission. Point B represents the peak amplitude. Point C present the ending time.

The parameters of the optical waveform are defined as follows. Rise-time (T_{AB}): Time of rising transition from the zero-reference level moment to the peak amplitude. Duration (T_{AC}): Time difference between the zero-reference level moments to the final transition of pulse (overshoot). Signal-to-noise ratio (SNR): In order to eliminate the influence of subsequent waveform, we use the quantity *Pre_SNR*, which is defined by the following equation[1,2].

$$Pre\_SNR = 10 \times log_{10} \frac{\sum_{peak-0.3ms}^{peak+4ms} Flux^2}{\sum_{peak-30ms}^{peak-0.3ms} Flux^2}.$$

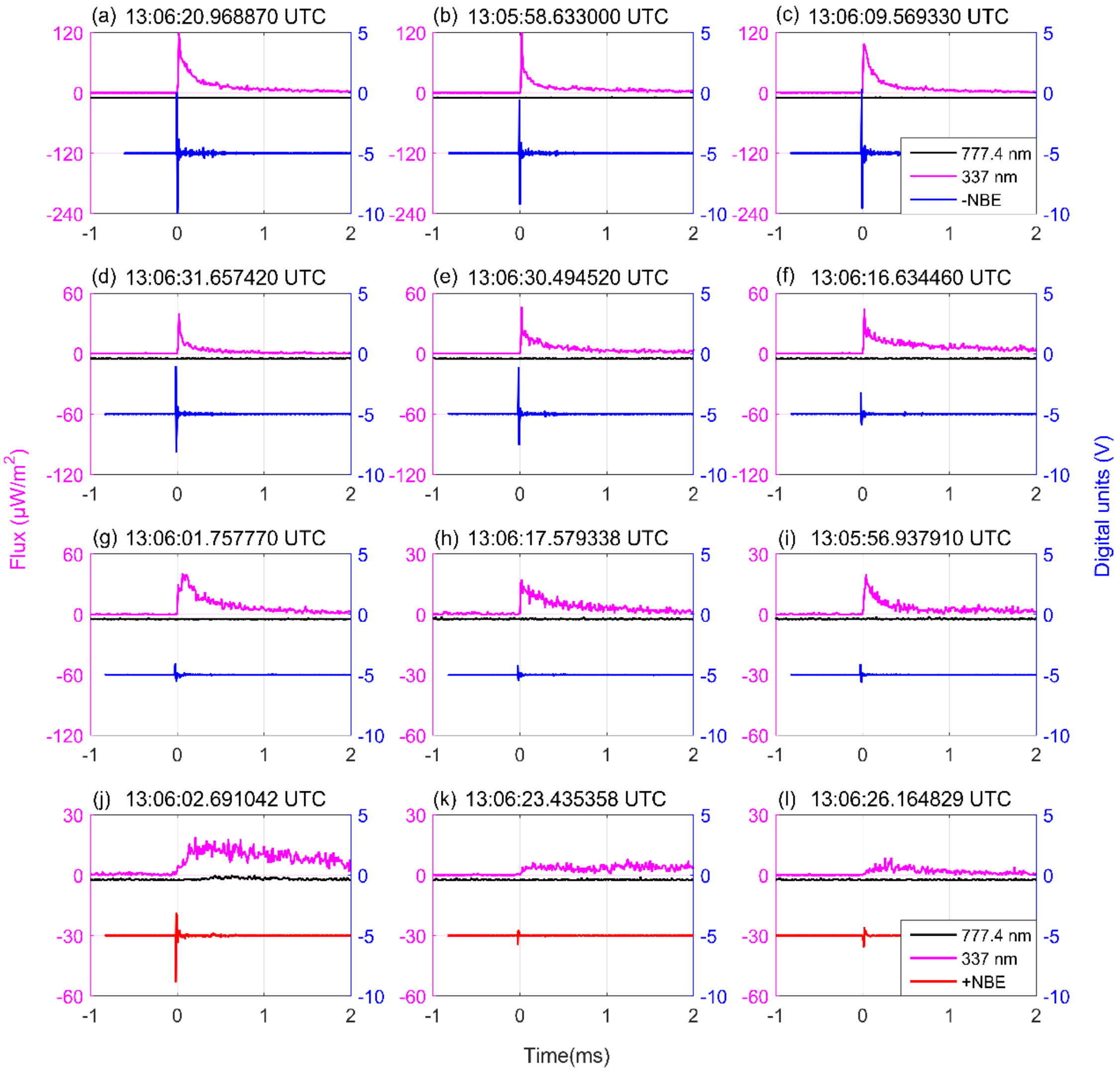

Supplementary Figure 4 | Optical and electrical signature of all NBEs: (a)-(i) negative NBEs; (j)-(l) positive NBEs. The VLF/LF waveform of negative NBEs is plotted with blue line, and that of positive NBEs is plotted with red line; the 337 nm and 777.4 nm emission are plotted with pink and black lines, respectively.

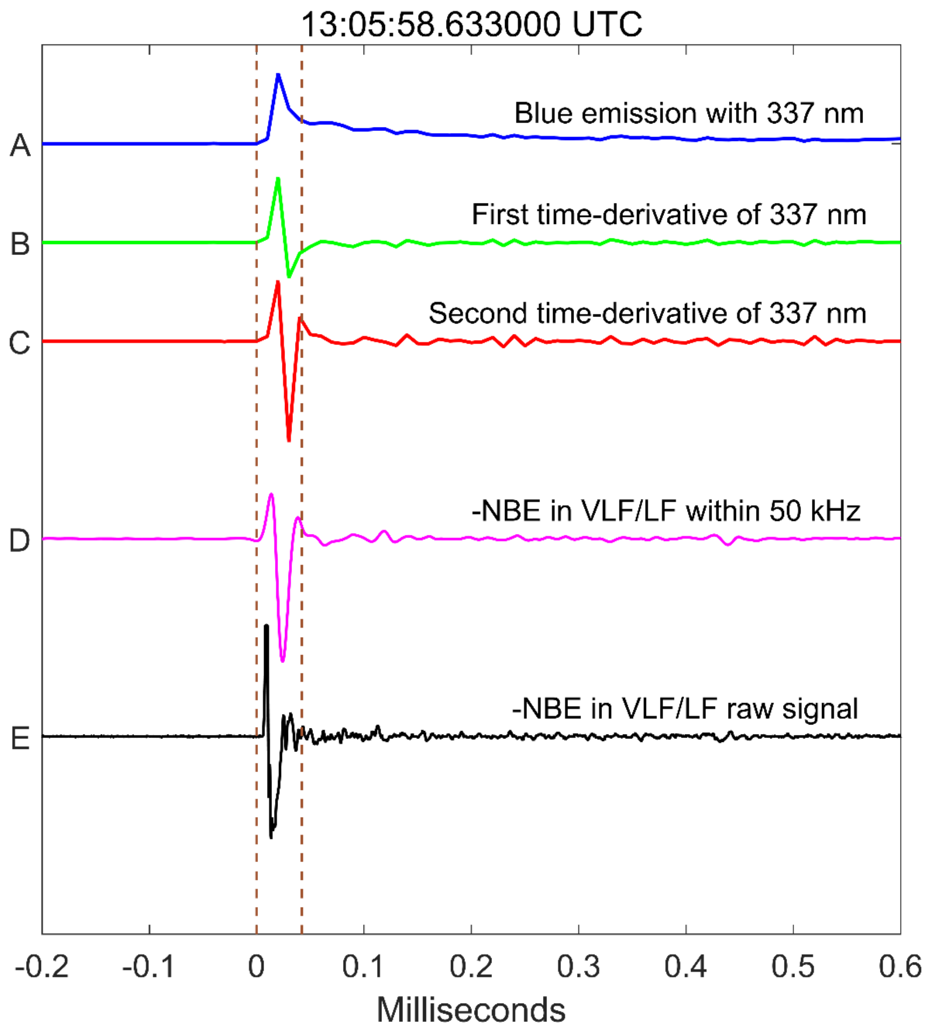

Supplementary Figure 5 | Comparison between VLF/LF signal within 50 kHz of negative NBE and the second time-derivative of blue emission. t = 0 ms corresponds to 13:05:58.633000 UTC on 7 August 2019.

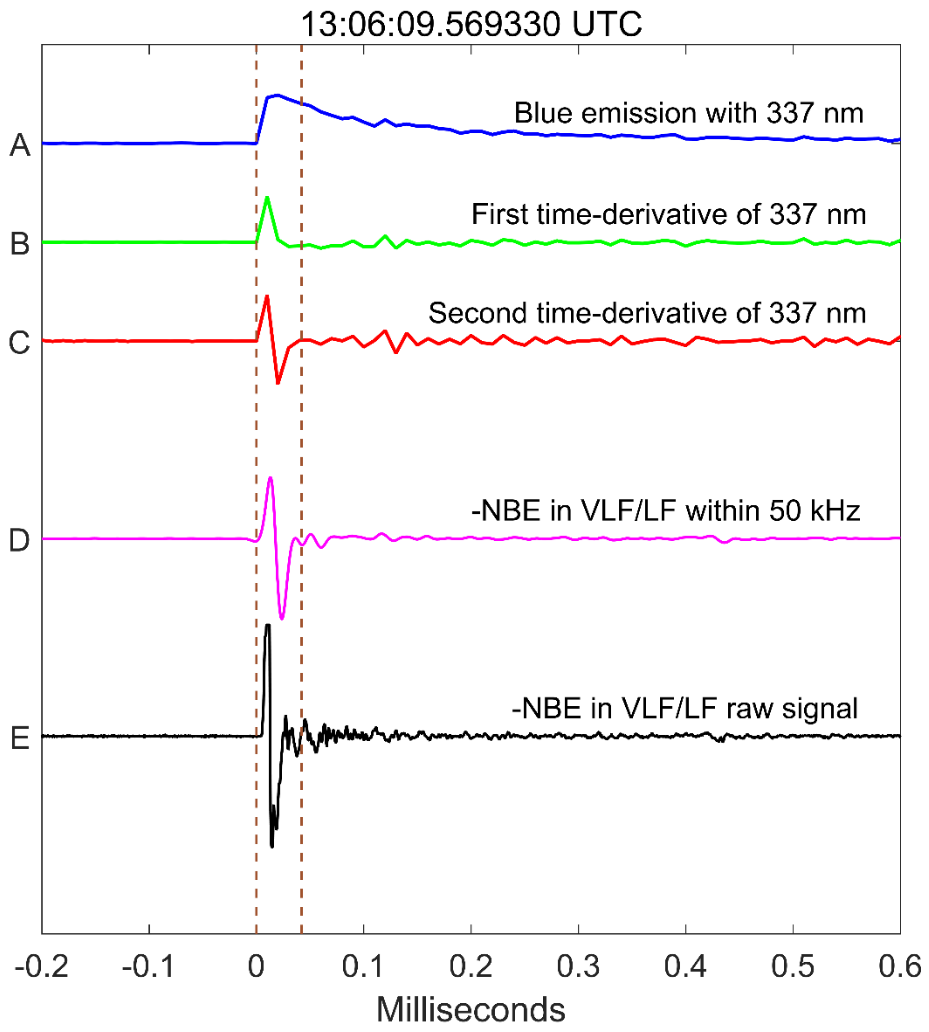

Supplementary Figure 6 | Comparison between VLF/LF signal within 50 kHz of negative NBE and the second time-derivative of blue emission. t = 0 ms corresponds to 13:06:09.569330 UTC on 7 August 2019.

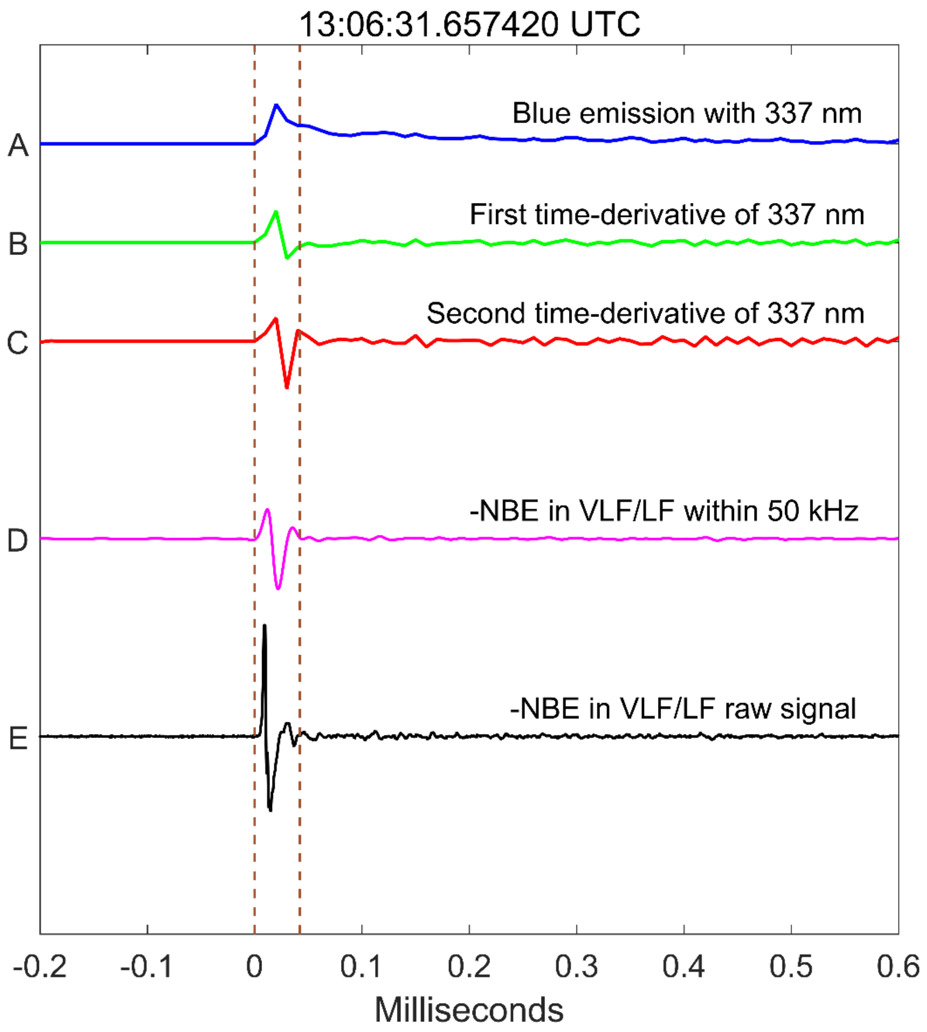

Supplementary Figure 7 | Comparison between VLF/LF signal within 50 kHz of negative NBE and the second time-derivative of blue emission. t = 0 ms corresponds to 13:06:31.657420 UTC on 7 August 2019.

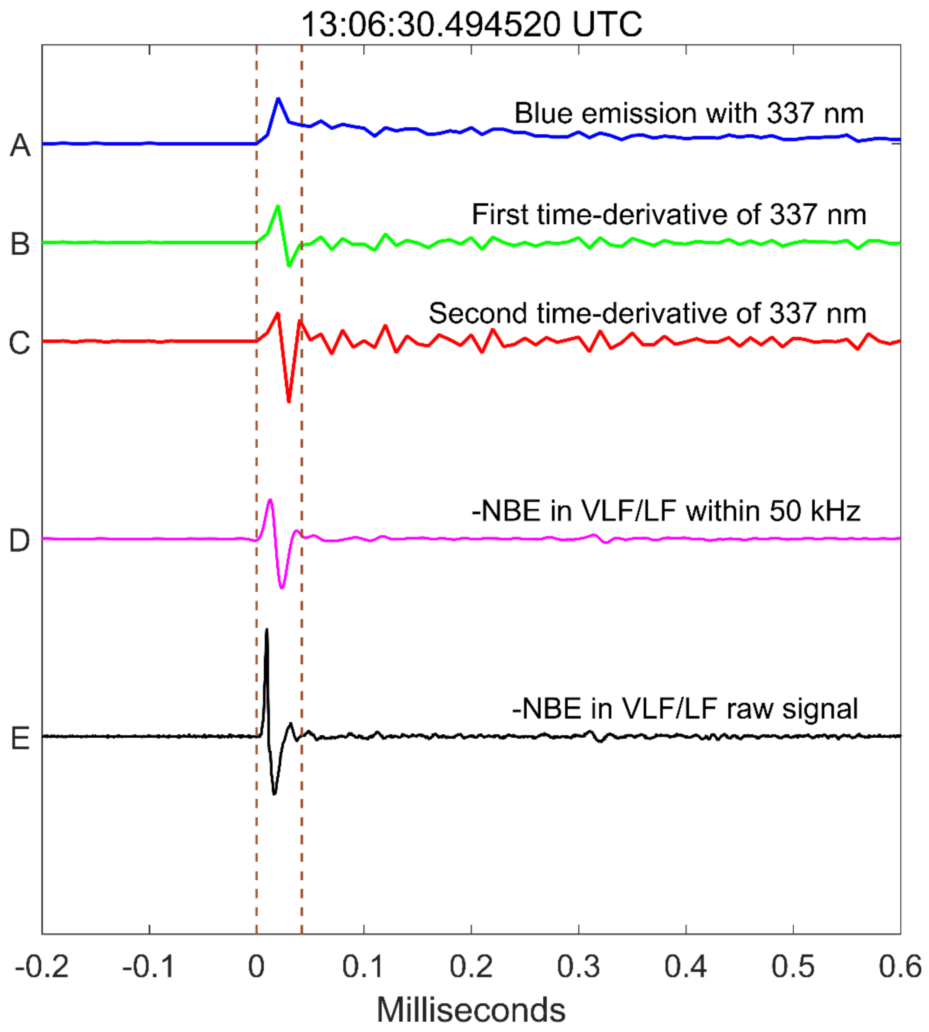

Supplementary Figure 8 | Comparison between VLF/LF signal within 50 kHz of negative NBE and the second time-derivative of blue emission. t = 0 ms corresponds to 13:06:30.494520 UTC on 7 August 2019.

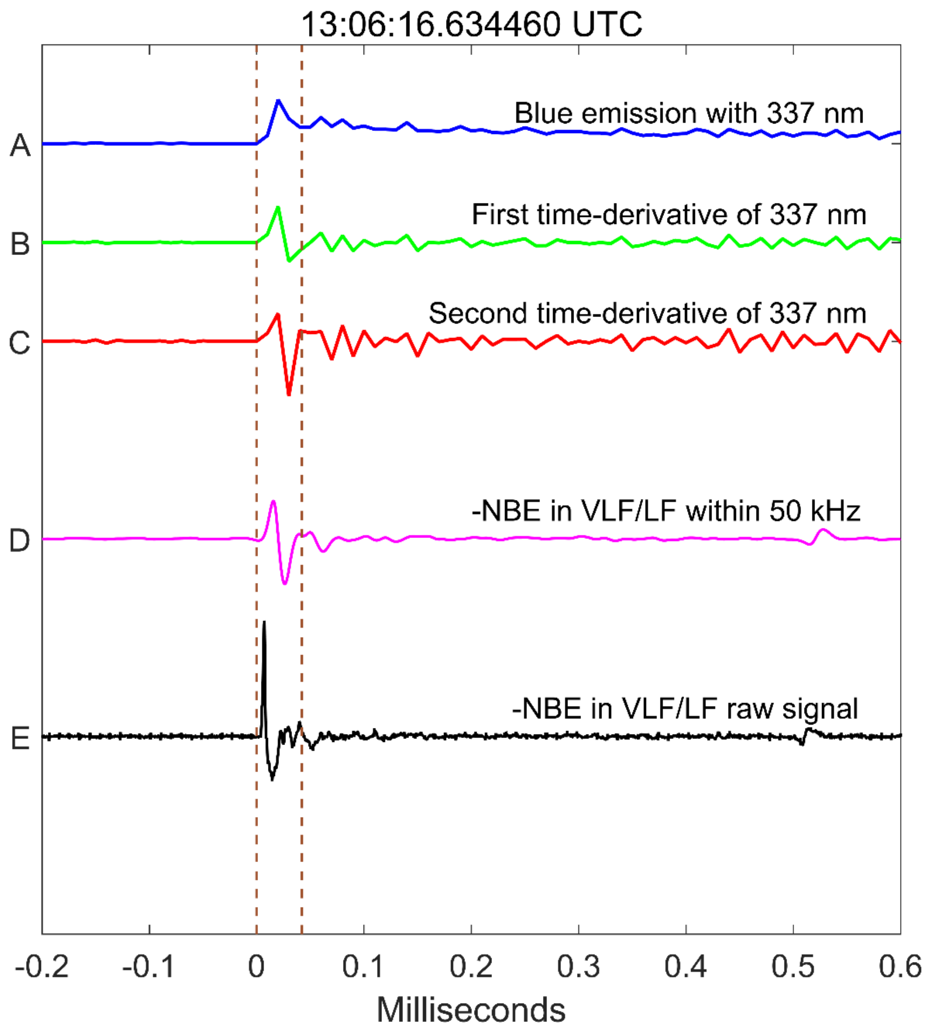

Supplementary Figure 9 | Comparison between VLF/LF signal within 50 kHz of negative NBE and the second time-derivative of blue emission. t = 0 ms corresponds to 13:06:16.634460 UTC on 7 August 2019.

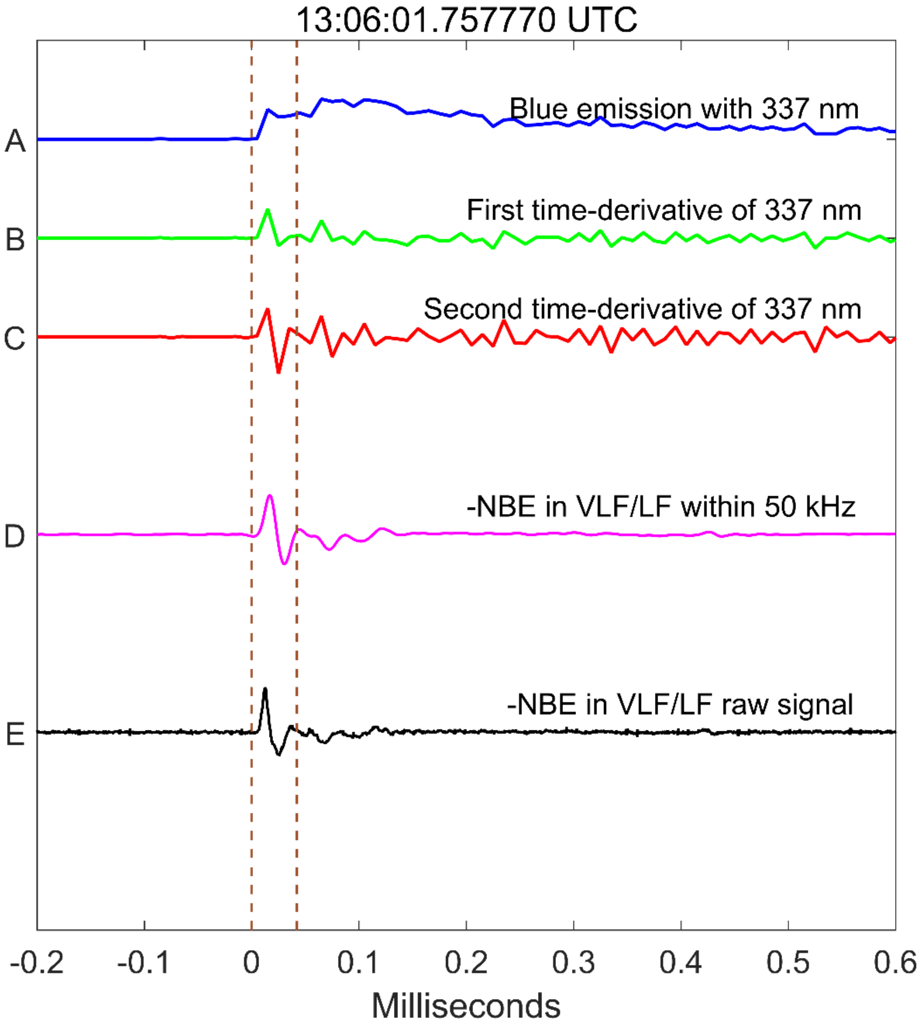

Supplementary Figure 10 | Comparison between VLF/LF signal within 50 kHz of negative NBE and the second time-derivative of blue emission. t = 0 ms corresponds to 13:06:01.757770 UTC on 7 August 2019.

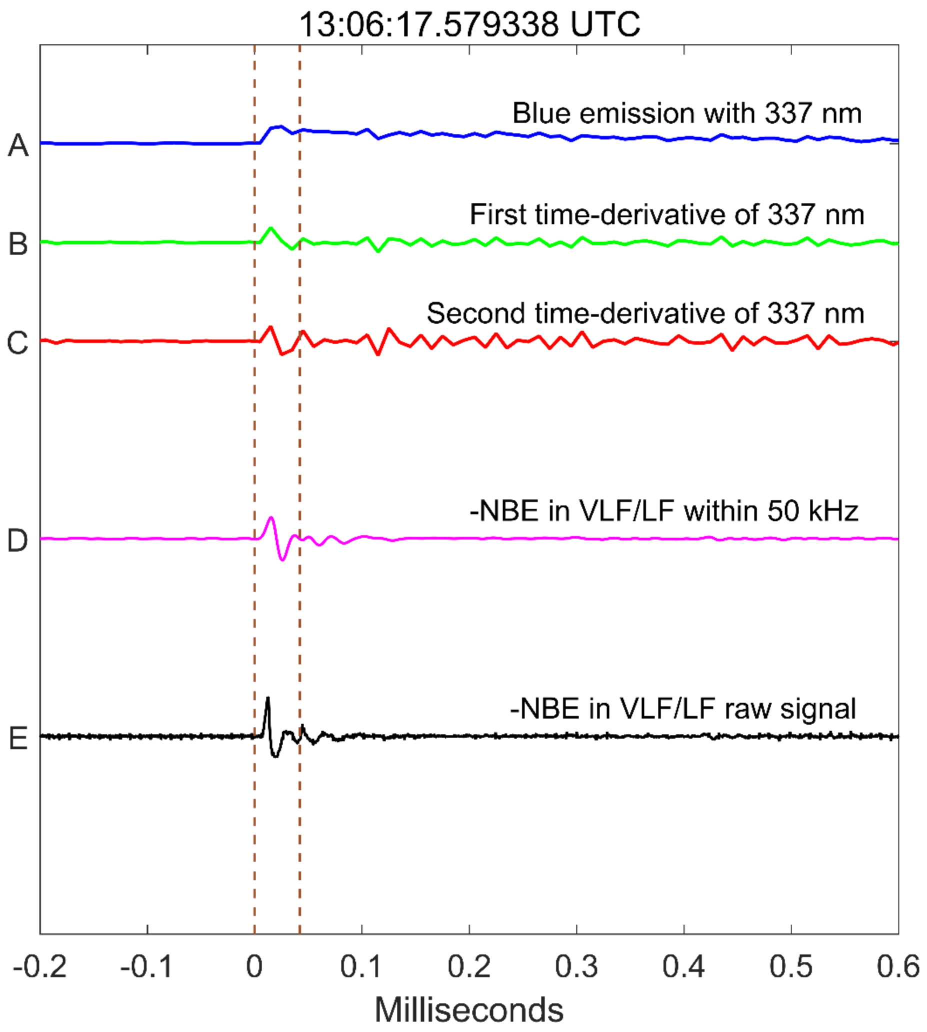

Supplementary Figure 11 | Comparison between VLF/LF signal within 50 kHz of negative NBE and the second time-derivative of blue emission. t = 0 ms corresponds to 13:06:17.579338 UTC on 7 August 2019.

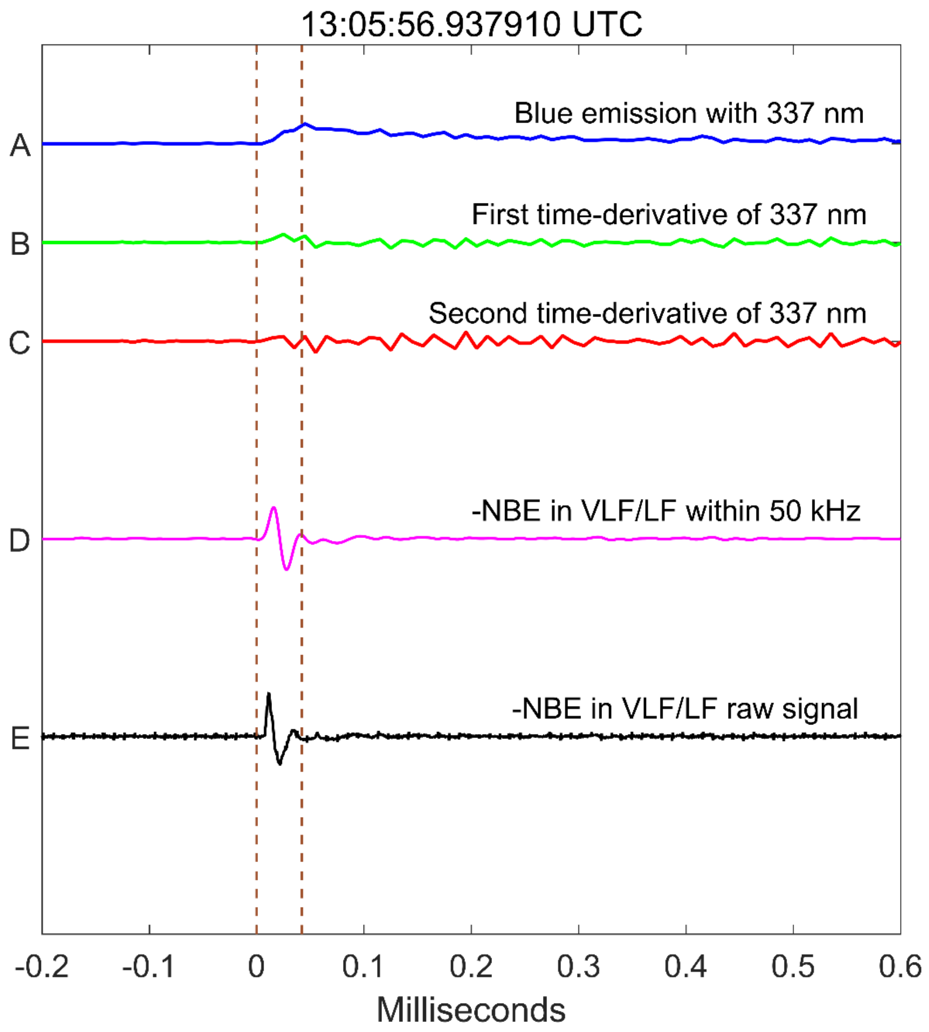

Supplementary Figure 12 | Comparison between VLF/LF signal within 50 kHz of negative NBE and the second time-derivative of blue emission. t = 0 ms corresponds to 13:05:56.937910 UTC on 7 August 2019.

Supplementary Table 1 | Time match information of blue events with ground-based VLF receiver.

Date	ASIM Optical Trigger Time (UTC)	Time Shift (ms)	VLF Trigger Time (UTC)	Type
2019/8/7	13:05:56.937910	+22 ms	13:05:56.9597594	-NBE
2019/8/7	13:05:58.633000	+22 ms	13:05:58.6551992	-NBE
2019/8/7	13:06:01.757770	+22 ms	13:06:01.7803276	-NBE
2019/8/7	13:06:02.691042	+21 ms	13:06:02.7128139	+NBE
2019/8/7	13:06:09.569330	+3 ms	13:06:09.5725785	-NBE
2019/8/7	13:06:16.634460	+4 ms	13:06:16.6388164	-NBE
2019/8/7	13:06:17.579338	+4 ms	13:06:17.5838553	-NBE
2019/8/7	13:06:20.968870	+4 ms	13:06:20.9729968	-NBE
2019/8/7	13:06:23.451358	+4 ms	13:06:23.4563116	+NBE
2019/8/7	13:06:26.164829	+4 ms	13:06:26.1689388	+NBE
2019/8/7	13:06:30.494520	+5 ms	13:06:30.4996518	-NBE
2019/8/7	13:06:31.657420	+5 ms	13:06:31.6619366	-NBE

Reference

1. Hamlin T., T. E. Light, X. M. Shao, K. B. Eack, and J. D. Harlin. Estimating lightning channel characteristics of positive narrow bipolar events using intrachannel current reflection signatures, *J. Geophys. Res. Atmos*, 115 D20 (2007).
2. Smith, D. A., Eack, K. B., Harlin, J., Heavner, M. J., Jacobson, A. R., Massey, R. S., Shao, X. M., and Wiens, K. C., The Los Alamos Sferic Array: A research tool for lightning investigations, *J. Geophys. Res. Atmos*, 107 D13 (2002).
